# Do medical residents perform patient-centered medical home tasks? A mixed-methods study

**DOI:** 10.1080/10872981.2017.1352434

**Published:** 2017-07-31

**Authors:** Lauren Block, Nancy LaVine, Jennifer Verbsky, Ankita Sagar, Miriam A. Smith, Susan Lane, Joseph Conigliaro, Saima A. Chaudhry

**Affiliations:** ^a^ Department of Medicine, Northwell Health, Lake Success, NY, USA; ^b^ Department of Medicine, Stony Brook University Medical Center, Stony Brook, NY, USA; ^c^ Office of Academic Affairs, Memorial Healthcare System, Hollywood, FL, USA

**Keywords:** Graduate medical education, patient-centered medical home, entrustable professional activities, nominal group technique, team-based care, care coordination

## Abstract

**Background**: Increasingly, residents are being trained in Patient-centered Medical Home (PCMH) settings. A set of PCMH entrustable professional activities (EPAs) for residents has been defined but not evaluated in practice.

**Objective**: To understand whether residents trained at PCMH sites reported higher likelihood of engaging in PCMH tasks than those training in non-PCMH sites.

**Design**: Survey and nominal group data from post-graduate trainees at three residency programs.

**Results**: A total of 179 residents responded (80% response). Over half (52%) cared for patients at PCMH sites. Residents at PCMH sites were more likely to report engaging in tasks in the NCQA domains of enhancing access and continuity (p < 0.01 for 4/11 tasks), planning and managing care (p < 0.01 for 3/4 tasks), providing self-care and community support (p < 0.01 for 3/5 tasks), and identifying and managing patient populations (p < 0.01 for 1/6 tasks), but were not more likely to report tracking and coordinating care or measuring and improving performance. Residents at PCMH sites were more likely to report working with medical assistants (p < 0.01), but not other healthcare professionals. Qualitative data showed staff teamwork and continuity of care as facilitators of patient-centered care, and technological problems and office inefficiencies as barriers to care.

**Conclusions**: Residents trained at PCMH sites were more likely to engage in tasks in several NCQA domains, but not care coordination and quality assessment. Similar facilitators and barriers to trainee provision of patient-centered care were cited regardless of PCMH status. Curricula on PCMH principles and workflows that foster continuity and communication may help to inform residents on PCMH tenets and incorporate residents into team-based care.

**Abbreviations**: EPA: Entrustable professional activity; GIM: General Internal Medicine; NCQA: National Center for Quality Assurance; PCMH: Patient-centered medical home

## Introduction

The patient-centered medical home (PCMH) model has been adopted by practices and health systems across the country as a way to provide care which better meets the needs of patients than traditional models [1]. PCMH models have been associated with improvements in patient satisfaction and provider burnout, as well as more modest changes in clinical outcomes [2–7]. One of the most common PCMH certifications is through the National Center for Quality Assurance (NCQA) Recognition Program, which is nearing the end of its first decade, having been established in 2008. The NCQA’s program has continued to adapt to current payment models and is launching its next version of certification in March 2017, to align with the shift to value based care through the Medicare Access and CHIP Reauthorization Act of 2015 (MACRA). The inclusion of trainees in these patient-centered medical home delivery systems is critical to developing and maintaining a robust primary care workforce.

Residents are commonly trained in ambulatory settings which incorporate PCMH principles and in NCQA-certified medical home practices [8]. Several professional organizations including the Alliance for Academic Internal Medicine advocate a move to training based on patient-centered care [9]. Recognizing that working in a PCMH does not necessarily equate with understanding PCMH goals and priorities, PCMH curricula for residents have been developed [10–12]. Several studies have described both faculty and resident knowledge and attitudes towards PCMH principles, as well as barriers to delivering patient-centered care in a training environment [13,14]. The performance of individual components of patient-centered care, including team-based care, group visits, and quality improvement, has been studied [15–18]. Little is known about how often residents perform the full scope of PCMH activities, as defined by a set of 25 PCMH Entrustable Professional Activities (EPAs) for residents [19].

We set out to examine how often residents perform patient-centered activities in practices with PCMH certification, compared with residents training in traditional practice models. We also examined whether residents in PCMH sites were more likely to report a favorable ambulatory experience. To improve our understanding of the facilitators and barriers to patient-centered care, we included a qualitative analysis of resident, faculty, and staff perceptions at one PCMH and one non-PCMH site. To minimize confounding, we included two residency programs in which residents had been assigned upon entering the program to either a PCMH or non-PCMH ambulatory clinic site.

## Methods

This mixed-methods study included a cross-sectional survey of internal medicine residents at three residency programs in New York in 2014. We also conducted a nominal group technique of faculty, staff, and residents at one of the sites. The primary outcome was resident-reported performance of PCMH tasks, derived from the PCMH EPAs defined by Chang et al. [19]. Secondary outcomes included self-reported learning and teaching, collaboration with the multi-disciplinary team, and satisfaction with the clinic. The study was approved by the Northwell Health and the Stony Brook University Hospital IRBs. Survey consent was provided by all survey participants; written consent was provided by focus group participants.

### Programs and ambulatory clinics

Table 1 lists characteristics of the eight ambulatory sites involved in this study. The internal medicine training program of Northwell Health (NW) is a University program comprised of 113 categorical residents assigned to two ambulatory sites. 70% are assigned to a combined faculty/resident practice which was awarded NCQA Level 3 PCMH status in 2009. The non-PCMH site is a hospital-based resident practice.

**Table 1.** Ambulatory site characteristics, internal medicine residency practices participating in a survey of patient-centered medical home entrustable professional activities.

	Site 1	Site 2	Site 3	Site 4	Site 5	Site 6	Site 7	Site 8
Program	NW^a^	NW	SBUH	SBUH	FHH	FHH	FHH	FHH
PCMH; year certified	No	Yes; 2009	Yes; 2014^b^	No	No	No	No	No
Description	Hospital	University	University	VA	University	Private practice	Private practice	Private practice
Residents per site	74	38	44	39	20	2	12	4
EMR; year introduced	Yes; 2012	Yes; 2010	Yes; 2010	Yes; 2000	Yes; 2014	No	Yes; 2011	Yes; 2000
Faculty preceptors	4	18	4	9	3	1	2	1
# professional disciplines	7	11	6	4	1	1	2	5
Schedule	4 + 1	4 + 1	4 + 1	4 + 1	Half-day	Half-day	Half-day	Half-day
# sessions per week	4–5	10	8	3–7	1	1	1	1

* Professional disciplines included registered nurse, medical office assistant; certified diabetes educator; social worker; pharmacist; health coach; registered dietician; licensed practical nurse; nurse practitioner; physician assistant; case manager; outreach coordinator

**All practices except the VA accepted Medicare, Medicaid, and uninsured patients

^a^ Resident only practice. All other practices included residents and faculty

^b^ Program contains a PCMH curriculum.

Forest Hills Hospital (FHH) is a community hospital within Northwell Health with an internal medicine residency program of 38 residents. FHH contains four ambulatory sites, all of which are non-PCMH private practice sites.

Stony Brook University Hospital (SBUH) is a University program of 83 residents at two ambulatory sites. A total of 53% are assigned to a PCMH site and the remainder to a VA site. The PCMH site received its NCQA level 3 accreditation in 2014, prior to distribution of the survey.

### Survey

We defined key PCMH tasks based on each of the 25 PCMH EPAs defined by Chang and colleagues and espoused by SGIM [19]. We evaluated how frequently residents reported performance of these tasks during their most recent week of ambulatory clinic given two of the three residency programs were on a 4 + 1 model of ambulatory education. These tasks were organized into six domains based on NCQA PCMH standards: (1) Enhance access and continuity, (2) Identify and manage patient populations, (3) Plan and manage care, (4) Provide self-care and community support, (5) Track and coordinate care, and (6) Measure and improve performance. Wording for each task was adapted and some EPAs split into multiple items based on pilot data on face and content validity with a group of chief residents and residents from other disciplines, for a total of 34 tasks pertaining to the 25 EPAs (see Table 2). Response choices included a 5-point Likert scale including ‘never’ = 1; ‘once per week’ = 2; ‘several times per week’ = 3; ‘daily’ = 4; ‘multiple times per day = 5.’

**Table 2.** Likelihood of performing PCMH activities in the most recent week of clinic as reported by residents*.

		PCMH Mean (SD)	Non-PCMH Mean (SD)	P
NCQA PCMH Standard 1: Enhance accessand continuity	Provided care between visits via:			
	1. Phone	3.2 (1.2)	2.2 (1.1)	<0.01
	2. Email	1.2 (.5)	1.1 (.3)	.23
	3. Remotely accessed EMR	2.0 (1.1)	1.4 (.7)	<0.01
	Group visits (any)	2.1 (.4)	2.1 (.5)	.31
	Accommodated care for patients with:			
	1. Language barriers	3.3 (1.3)	2.4 (1.3)	<0.01
	2. Cognitive barriers	2.3 (1)	2.3 (1)	.94
	3. Cultural barriers	2.9 (1.2)	2.5 (1.3)	.03
	Led team	1.9 (1.2)	1.9 (1.2)	.63
	Led huddle	2.3 (1.5)	2 (1.2)	.09
	Sought to improve care/access	2.7 (1.3)	2.5 (1.1)	.18
	Advocated for patients	3.6 (.9)	3.2 (1.2)	.008
NCQA PCMH Standard 2: Identify and managepatient populations	Considered practice needs	2.8 (1.2)	2.6 (1.2)	.19
	Intervened for patients with:			
	1. Functional impairment	2.8 (1.1)	2.5 (1.1)	.08
	2. Cognitive impairment	2.4 (1.1)	2.2 (1.0)	.29
	3. High risk meds	3.3 (1.1)	2.8 (1.1)	.01
	4. Chronic disease	3.9 (1)	3.5 (1.2)	.01
	5. Substance abuse	3.2 (1.1)	2.3 (1.1)	<0.01
NCQA PCMH Standard 3: Plan and manage care	Used EMR	4.8 (.5)	4.1 (1.4)	<0.01
	Developed long term care plan	4.1 (1)	3.8 (1.3)	.13
	Used guidelines	4.4 (.8)	3.8 (1.2)	.002
	Did medication reconciliation using EMR	4.8 (.5)	4.2 (1.3)	<0.01
NCQA PCMH Standard 4: Provide self-care andcommunity support	Counseled a patient on self-management	4.7 (.5)	4.1 (1.2)	<0.01
	Facilitated patient’s participation in own healthcare	4.5 (.7)	4.1 (1.2)	<0.01
	Did advance care planning	2.8 (1.5)	2.9 (1.4)	.71
	Advised on health behaviors	4.3 (.8)	3.9 (1.2)	.009
	Used community resources	3.5 (1)	3.1 (1.3)	.04
NCQA PCMH Standard 5: Track and coordinate care	Worked with clinic members to help patients attend visits	2.9 (1.4)	2.8 (1.3)	.46
	Worked with clinic to help patients transition	3 (1.3)	2.9 (1.2)	.56
	Coordinated care	3.2 (1.2)	3.1 (1.2)	.52
	Encouraged patients to track their own care	3.6 (1.2)	3.5 (1.2)	.60
NCQA PCMH Standard 6: Measure and improveperformance	Accessed data on clinic performance	2.1 (1.4)	2.5(1.4)	.03
	Engaged in QI	2.5 (1.4)	2.8 (1.2)	.17
	Used EMR to prevent errors	3.8 (1.4)	3.3 (1.5)	.03
	Studied sentinel event	2.1 (1.4)	2.1 (1.3)	.87

* All variables used a 5-point scale ranging from 1 = never to 5 = multiple times a day.

Secondary outcomes included measures of teaching and learning, satisfaction with clinic, and multi-disciplinary collaboration, a total of 43 items. We included composite indices of learning opportunities (nine items), faculty teaching (10 items), and staff roles (eight items) by Roth et al. [20]. We included two previously published items on likelihood of entering a general medicine career [21], one item on number of patients seen in a typical half-day session [22], and two items on satisfaction with the clinic [21,23] which used 5-point Likert scales ranging from ‘disagree strongly’ = 1 to ‘agree strongly’ = 5. The survey was pilot tested for face and content validity with five chief residents at one of the programs. Based on pilot data we added three demographic items and eight items on frequency of inter-professional collaboration (see Appendix).

The survey was distributed electronically to residents. No identifying information was shared with program directors. Respondents received no compensation.

### Nominal group technique (NGT)

To provide qualitative data regarding the facilitators and barriers to patient center care, we conducted a NGT at both Northwell ambulatory sites (the PCMH and non-PCMH hospital-based clinic). NGT provides semi-quantitative, rank-ordered feedback on participants’ perceptions of medical education programs [24]. The eight-step technique includes: presenting large group with evaluation question, small group formation to identify strengths/weaknesses, round robin phase with facilitator helping theme small group comments together, clarification phase, voting phase, small group data scoring, large group data combining, and wrap-up discussion. Large group data combining was omitted due to time constraints. The question posed to all participants was, ‘What are the barriers and facilitators to providing patient-centered care at the resident clinic?’ Faculty and staff meetings were held separately from resident meetings, and the meetings at the PCMH site were held separately from the non-PCMH site. Staff included social workers, pharmacists, medical assistants, nurses, nurse practitioners, and front desk staff. No compensation other than food was provided.

### Data analysis

Responses with Likert scale responses were analyzed as continuous variables and means compared between PCMH and non-PCMH sites. Quantitative data was analyzed using Stata 11IC (College Station, TX) using t-tests for continuous outcomes and chi-square tests for categorical outcomes. Due to multiple comparisons we used p < 0.01 to establish statistical significance. NGT data was tallied and presented as voted on by group participants.

## Results

A total of 221 residents were sent the survey and 179 responded, an overall response rate of 80% (Table 3). A total of 89 (83%) residents at Northwell, 61 (74%) at SBUH, and 29 (83%) at FHH responded. Gender, PGY, and clinic site among respondents were not significantly different from those of the overall programs. Overall 52% of residents saw patients in PCMH sites.

**Table 3.** Participant and practice characteristics at PCMH and non-PCMH residency clinic sites.

Variable		N	PCMH† N (%)	Non-PCMH N (%)	P Value
Total		179	95	84	
Residency	NW+	89	62 (70%)	27 (30%)	
	FH	29	0	29 (100%)	
	SBUH	61	33 (54%)	28 (46%)	
PGY year	1	60	30 (50%)	30 (50%)	.54
	2	59	32 (54%)	27 (46%)	
	3+	60	33 (55%)	27 (45%)	
Gender	M	98	52 (53%)	46 (47%)	.87

+ SBUH = Stony Brook University Hospital. NW = Northwell Health. FHH = Forest Hills Hospital.

† PCMH = Patient-Centered Medical Home.

Average number of patients seen per half day did not differ significantly between PCMH and non-PCMH. A minority of residents reported being ‘very likely’ to enter a GIM field before residency (10%) or at present (14%) and likelihood of entering GIM did not vary by PCMH assignment (data not shown).

### Performance of PCMH tasks

#### NCQA domain 1: enhance access and continuity

In the NCQA domain of enhancing access and continuity, residents trained at PCMH sites were more likely to report four of eleven tasks at p < 0.01, including providing care between visits via phone and electronic health record (EHR) accessed remotely (3.2 vs. 2.2 and 2.0 vs. 1.4, respectively, on a 1–5 Likert scale), accommodating care for patients with language barriers (3.3 vs. 2.4), and advocating for patients (3.6 vs. 3.2) (Table 2). Residents at PCMH sites were not more likely to report communicating with patients via email, participating in group visits, leading a team, leading a huddle, or seeking to improve care or access at the site.

#### NCQA domain 2: identify and manage patient populations

For the NCQA domain of identifying and managing patient populations we found one of six tasks to be significant at p < 0.01. At PCMH sites, residents reported intervening more frequently in patients with substance abuse (3.2 vs. 2.3, p < 0.01) and tended to intervene more frequently in patients with chronic diseases and high-risk medications (3.3 vs. 2.8; 3.9 vs. 3.5; both p = 0.01), but not patients with functional or cognitive impairments.

#### NCQA domain 3: plan and manage care

For the domain of planning and managing care, we found three of four tasks to be significant at p < 0.01. At PCMH sites, residents reported using the EHR in patient care (4.8 vs. 4.1), using guidelines (4.4 vs. 3.8), and performing medication reconciliation using the EHR (4.8 vs. 4.2) more frequently than at non-PCMH sites, but were not more likely to have developed a long-term care plan with their patients.

#### NCQA domain 4: provide self-care and community support

In providing self-care and community support, we found three of five tasks to be significantly different between the PCMH and non-PCMH sites. Residents at the PCMH sites were more likely to report counseling patients on self-management (4.7 vs. 4.1, p < 0.01), facilitating the patient’s participation in own healthcare (4.5 vs. 4.1, p < 0.01), and advising patients on health behaviors (4.3 vs. 3.9, p < 0.01), but were not more likely to use community resources or do advance care planning.

#### NCQA domain 5: track and coordinate care

Residents at PCMH sites were not more likely to help transition patients, coordinate care, or encourage patients to track their own healthcare.

#### NCQA domain 6: measure and improve performance

Residents at PCMH sites were not more likely to use the EHR to prevent medical errors, access data on clinic performance, engage in QI, or study sentinel events.

### Teaching and learning; satisfaction with clinic

As shown in Table 4, there was no difference in satisfaction with the ambulatory experience nor in ratings on the teaching or learning scales between PCMH and non-PCMH sites.

**Table 4.** Resident satisfaction with clinic, teaching and learning, and collaboration.

Domain	Question stem	Item	PCMH	Non-PCMH	p-value
Satisfaction	To what extent do you agree or disagree:	GIM enjoyable	3.7 (1.2)	4.0 (1.1)	.12
		Satisfied with ambulatory experience	3.8 (1.1)	3.9 (1)	.52
Teaching and learning	Faculty teaching†		4.0 (1.6)	3.7 (1.8)	.33
	Learning opportunities		3.4 (1.5)	3.3 (1.8)	.67
Interprofessionalcollaboration	Staff roles		3.0 (1.4)	3.1 (1.6)	.63
	How often did you do the following inthe most recent week of clinic?	Worked with social workers	2.9 (1.3)	2.6 (1.3)	.07
		Worked with nurses	4.1 (1.1)	3.9 (1.3)	.32
		Worked with pharmacists	2.4 (1.3)	2.8 (1.4)	.06
		Worked with nutritionists	2.1 (1.2)	2.2 (1.3)	.76
		Worked with diabetes educators	2.3 (1.3)	2.2 (1.2)	.66
		Worked with NPs	2.5 (1.5)	2.0 (1.4)	.045
		Worked with case managers	1.9 (1.2)	1.9 (1.2)	.63
		Worked with medical assistants	4 (1.4)	3.3 (1.6)	<0.01

* All variables other than learner, staff, and faculty scales used a 5-point scale ranging from 1 = very unlikely to 5 = very likely.

† Faculty, learner, and staff scales are composite scales consisting of 10, 9, and 8 items respectively.

### Inter-professional collaboration

There were no significant differences in ratings on the staff scales. Residents at PCMH sites were more likely to report working with medical assistants (4.0 vs. 3.3, p < 0.01), but were not significantly more likely to report working with NPs, social workers, nurses, pharmacists, nutritionists, diabetes educators, or case managers even though many of these professionals worked at the PCMH practices (Table 4).

### Qualitative data

Table 5 lists the top three facilitators to providing patient-centered care at the PCMH as reported by residents, which were knowledgeable faculty, continuity of care, and clinic resources, including POC testing, diabetes educators, and dieticians. At the non-PCMH site, the top three facilitators listed by residents were the nurse and resident-led triage system, continuity of care, and ancillary staff. At the PCMH site, the top three facilitators reported by faculty and staff included the multidisciplinary team, continuity of care, and the pre- and post-visit huddles. At the non-PCMH site, the top three facilitators reported by faculty and staff included staff teamwork, continuity of care, and the EMR.

**Table 5.** Top three facilitators of patient-centered care at PCMH and non-PCMH sites as reported by residents, faculty, and staff based on nominal group technique results.

PCMH	Non-PCMH
Facilitators	
Residents	Residents
1. Faculty are knowledgeable, teach, and don’t micromanage	1. Triage system
2. Continuity of patient care improving/rewarding as a physician	2. Improved continuity of care
3. Resources at clinic (POC testing, diabetes educators, dieticians)	3. Ancillary staff provide support
Faculty/staff	Faculty/staff
1. Extensive multidisciplinary team that works to the highest level of their license	1. Staff excellence and teamwork
2. Continuity strategies	2. Continuity and resident scheduling
3. Pre-visit and post-visit assessment of patient needs	3. EHR
Barriers	
Residents	Residents
1. Scheduling (inflexible, patient delays, provider delays, lose continuity)	1. EMR is cumbersome and complicated
2. Technology (computer, EMR, phones)	2. Office efficiency
3. Secretarial	3. Patient registration is messed up
Faculty/staff	Faculty/staff
1. Quality of EMR and other technological issues	1. Space
2. Communication between staff members	2. Registration/scheduling
3. Poor continuity with patients on controlled substances	3. Staffing (lack of)

The top three barriers to providing patient-centered care at the PCMH as reported by residents were inflexible scheduling and delays, technological problems with the EMR and phone system, and lack of secretarial services for the resident clinic. At the non-PCMH site, the top three barriers listed by residents were the cumbersome EMR, lack of office efficiency, and problems with patient registration. At the PCMH site, the top three barriers reported by faculty and staff included the EMR quality and other technological problems, communication between staff members, and poor continuity with patients on controlled substances. At the non-PCMH site, the top three barriers reported by faculty and staff included lack of space, slow registration, and lack of staff.

## Discussion

As more ambulatory care moves to a PCMH model, we can expect an increasing number of residents training in PCMH sites. The residents we surveyed reported caring for high-risk patients, including those with language barriers, chronic diseases, and those on high-risk medications. Residents at PCMH sites were more likely to report engaging in several PCMH tasks required to provide quality care to these high-risk patient populations, including enhancing access and continuity, identifying and managing patient populations, planning and managing care, and providing self-care and community support through such activities as using the EMR, performing medication reconciliation, and advocating for patients.

Training in a PCMH may not, however, equate with participation in all PCMH activities. Residents at PCMH sites were not more likely to report tracking and coordinating care, key features of population management. Residents at PCMH sites were more likely to work with medical assistants but reported working with other professionals such as social workers and diabetes educators infrequently even though these professionals were employed by these PCMH sites. Opportunities for collaboration between these professionals are facilitated at several of our sites by shared inboxes and team-based care coordination workflows to ensure prompt follow-up for abnormal results and for patients accessing emergency and hospital services. The lack of frequency with which residents reported working with these professionals despite these opportunities signals an opportunity to expand collaboration and clarify roles of the multi-disciplinary team. Lack of communication within the multidisciplinary team was cited as a barrier to patient-centered care by both residents and faculty/staff in the focus groups. Expanding access to these professionals for residents and creating workflows that facilitate communication may promote collaboration and team functioning [25]. In both 4 + 1 and traditional scheduling models, consideration must be paid to ensuring effective communication and teamwork given residents spend only 10–20% of their time in the ambulatory clinic.

Residents at PCMH sites were not more likely to report performing tasks in the domain of measuring and improving performance. Quality improvement (QI) was reported infrequently by residents at all sites despite the fact that residents are required to conduct two quality improvement projects per the ACGME. Residents may not equate participation in these projects with QI. Orientation to the QI process, peer feedback, inclusion of residents in PDSA cycles, and encouraging resident teams to present QI projects are several ways to promote resident engagement in QI [16,26–28].

One potential reason residents do not report engaging in all aspects of patient-centered care may be limited insight into the functioning of the PCMH. At the institutions we surveyed, no formal PCMH curriculum exists. More residency programs are now incorporating training in PCMH principles for medical students and residents, but such training is not universal [10–12]. Moreno and colleagues found that family and internal medicine residents wanted additional learning about the PCMH [29]. Similarly, El Rayess and colleagues found that while residents and faculty at one level 3 PCMH had positive attitudes toward the PCMH, both residents and faculty reported lacking knowledge and feeling unprepared for PCMH activities [13]. When the same group added a PCMH block rotation and didactic curriculum, the intervention group reported improved PCMH knowledge, skills, and experience from ‘basic’ to ‘intermediate’ level [30]. Lee recommended including education in care management, teamwork, and community resources in training residents for careers in new practice models [31].

The timing of PCMH transformation may impact resident workflows and activities. A strength of our study was inclusion of PCMH sites at different stages of development. At Stony Brook, for instance, the resident clinic was certified by the NCQA as a PCMH only one month prior to the survey; workflows and processes may not have been fully established at the time of survey completion. Alternatively, residents who witnessed the PCMH transformation may have been more aware of changes taking place than residents who had limited involvement in the initial PCMH application at Northwell.

Qualitative data confirmed that continuity and teamwork were goals at all sites and that technological and office efficiency problems hindered provision of patient-centered care, signaling an opportunity to improve continuity in resident practices. A relatively new EMR at all sites brings the opportunity to improve efficiency in registration and visits through multi-disciplinary collaboration, provided adequate training and use of the EMR occurs [32].

Residents at the PCMH did not report higher satisfaction with ambulatory clinic and were not more likely to go into general medicine than residents at non-PCMH sites. While prior research has shown that factors other than the ambulatory experience shape career choices, improving certain aspects of communication, empanelment, mentoring, and continuity may impact overall satisfaction with the ambulatory experience and ultimately the primary care workforce [33,34].

Strengths of our approach include the fact that we studied three residency programs with vastly different ambulatory settings, a rarity in medical education research. We included PCMH, VA, hospital-based, and private practice sites. We evaluated PCMH sites in various stages of development, increasing applicability of our results to PCMH sites in a variety of stages of adoption, from the period of initial transformation to clinics sustaining a longstanding PCMH model. Our studies incorporated both quantitative data and resident, faculty, and staff voices in the qualitative data. We achieved a response rate of 80% and used validated measures of teaching and learning, satisfaction, and collaboration used in previous studies.

Limitations in this study included a limited sample size and geographic area. We minimized this by including three residency programs at two hospital systems, but the results may not be generalizable to other areas or specialties. Our survey was a cross-section, so causation cannot be implied by any association. Our survey included only residents, and experiences of other stakeholders, including faculty and staff, may be distinct. Baseline differences in patient demographics and socio-economic factors between sites, rather than different care models, may contribute to differences seen. Differences in frequency of activities is limited by the fact that the number of clinic sessions per week and number of patients seen in each clinic session varies by site, as shown in Table 1. Differences between groups of residents and programs are potential confounders, which are limited by the fact that residents are distributed between clinic sites before residency begins. Qualitative data shed light on quantitative responses and included the faculty and staff voice, but was only conducted at one of the three programs due to budget and time constraints. In addition, self-reported performance of tasks during a given week may not be representative of actual performance nor of proficiency at performing these tasks, and activities may vary throughout the academic year.

A PCMH model offers the promise of improved access and continuity but requires effort on the part of staff, faculty, and the organization to create new workflows and build team-based models incorporating residents [35]. While some aspects of patient-centered care seem to be quickly taken up by residents practicing at PCMH sites, others including population management and quality improvement may require more training and faculty participation. Based on several factors including the results of this study, Stony Brook implemented team-based quality improvement projects involving residents and nursing staff in the management of atrial fibrillation and chronic pain. Northwell Health is moving towards a ‘teamlet’ clinic structure to incorporate medical assistants and nurses more fully into resident teams, with the goal of improving the management of population health through pre-visit planning and team-based huddles. Forest Hills is planning a transition to an ‘x+y’ resident ambulatory experience including dedicated time for quality improvement projects. Next steps will include an assessment of resident, staff, and faculty engagement in PCMH tasks as these and other clinical transformations proceed. Involving residents in the process of clinical and educational transformation and team-based QI projects may help residents engage fully in the PCMH model and bring patient-centered care to their patients.
